# The complete plastome of *Rorippa palustris* Besser 1821 and its phylogenetic analysis

**DOI:** 10.1080/23802359.2024.2406929

**Published:** 2024-09-25

**Authors:** Xinhua Wang, Liqiang Wang, Jiaojiao Kong, Hongqin Li, Na Kong

**Affiliations:** a College of Pharmacy, Heze University, Heze, Shandong Province, P. R. China; b Personnel Office, Heze Medical College, Heze, Shandong Province, P. R. China

**Keywords:** Brassicaceae, plastome, Rorippa palustris, phylogenetic analysis

## Abstract

*Rorippa palustris* Besser 1821, a species of Brassicaceae, is widely distributed around the world and used for both food and traditional Chinese medicinal purposes. Despite the plant’s significance, its genetic diversity must be better understood. In this study, we have successfully assembled and characterized a complete plastome of *R. palustris*, marking a significant advancement toward comprehending its genetic composition. The plastome is 154,674 bp long and harbors 128 genes, including 83 protein-coding genes, 37 tRNA genes, and eight rRNA genes. Our phylogenomic analysis indicated that *R. palustris* is closely related to *R. curvipes*. These findings are crucial for conserving and utilizing this important plant species. They also highlight the potential for future research into the evolution and preservation of *R. palustris*, which could be advantageous in pharmaceutical applications.

## Introduction

Rorippa, a genus within the Brassicaceae family, comprises 75 species of herb plants in the Northern Hemisphere’s warm temperate zone (Zheng et al. 2021), with 11 species in China (Wang, 2022). The model species of Rorippa is Rorippa amphibia Besser 1821, characterized by features such as typically living in a humid or aquatic environment, one-year-old to perennial herbs with one delicate taproot, upright stems, plumose deeply lobed or nearly entire leaves, and yellow petals as long as sepals (Zhang et al. 2009). These plants have a wetland habit and tend to flower from April to July and bear fruit from June to August (Li et al. 2023).

*Rorippa palustris* Besser 1821 (Besser 1821), a member of the *Rorippa* genus, is widely distributed in the warm temperate zone and is used as a food plant and in traditional Chinese medicine (TCM). Its tender stems and leaves can be eaten (Tian and Cheng. 2003). In field investigations, local residents often refer to it as “water shepherd”, a nutrient-rich wild vegetable. *Rorippa palustris* stands out from other wild vegetables due to its high concentration of organic acid and soluble sugar (Wang et al. 2022, Tian and Cheng 2003). The whole grass is employed in TCM under the name Rorifone, prescribed for conditions such as wind-heat colds, sore throats, jaundice, edema, and arthritis. These compounds have demonstrated cough-relieving and phlegm-expelling properties (Chinese Herbal Medicine Color Map, 2020 edition), antibacterial effects, anti-inflammatory effects, relieving exterior symptoms and dispelling colds, promoting blood circulation and detoxification, removing dampness and curing jaundice (Chinese Pharmacopeia, 2020 edition). Despite its pharmaceutical potential, the genetic diversity of *R. palustris* remains relatively unexplored. It has been demonstrated in several research studies that plastome genome sequences play an essential role in plant phylogeny and genetic analysis (Ma et al. 2021; Yu et al. 2021).

Therefore, this study aimed to assemble and characterize the complete plastome of the plant, which could serve as a foundation for future research on the evolution, conservation, and potential medicinal applications of *R. palustris*.

## Materials

**Figure 1.**
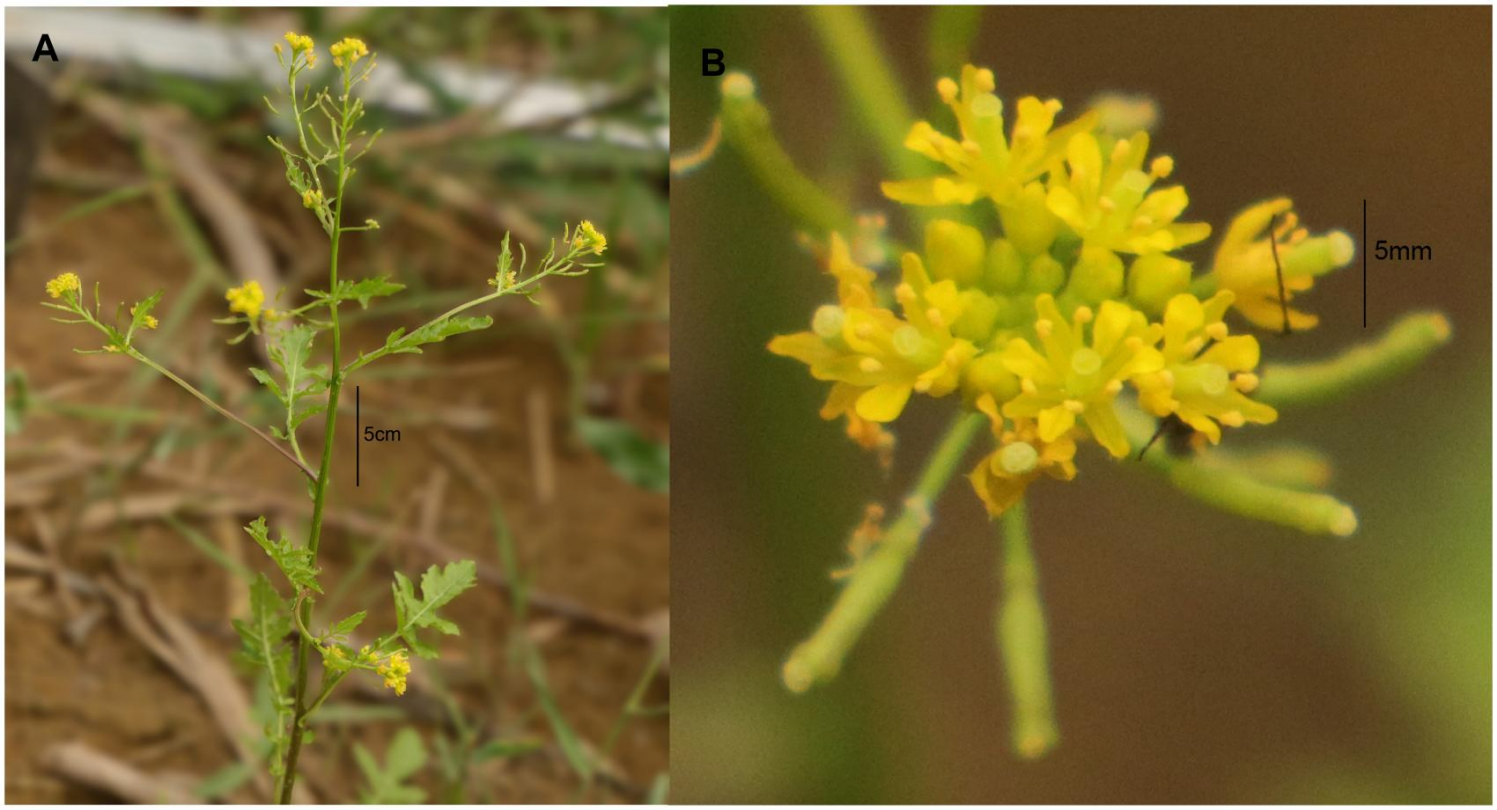
Field photo of *Rorippa palustris*. The author Liqiang Wang took the photo at the position of 35° 15’ 9.27’’ N, 115° 29’ 44.44’’E. Main identifying traits: the basal leaves are petiolate, pinnately lobed and oblong to narrowly oblong; the cauline leaves are nearly sessile and deeply feathered. The botryose inflorescences are terminal or axillary, bractless. Elongating clusters at the end of branching stems and arising from the leaf axils, with a small compact flower head at the tip and fruit developing below it. Flowers are across with 4 yellow spatula-shaped petals about 2 mm long alternating with 4 yellowish-green oblong sepals that are about as long as the petals. 6 yellow stamens and a stout style are in the center. The short-horned fruit is nearly cylindrical or elliptic. The seeds are small, nearly ovate and flat. Scale bar in (A) is shown as 5 cm; scale bar in (B) is shown as 5 mm.

The fresh leaves used for sequencing were obtained from the Peony District, Heze City, Shandong Province, China (35°15’9.27′’N, 115°29’44.44′’E) (Figure 1), and a sample has been deposited in the Heze University Herbarium (contact person: Hongqin Li, 463056627@qq.com) under the sample number HZ20220831. Genomic DNA was extracted using the plant genomic DNA kit (Tiangen Biotech, Beijing, China).

## Methods

The genomic DNA was carefully fragmented into roughly 300 bp segments to construct sequencing libraries. These libraries were then sequenced using the state-of-the-art Illumina NovaSeq 6000 platforms at Wuhan Benagen Technology Company Limited, located in Wuhan, China. The raw sequencing data underwent a precise trimming process using Trimmomatic 0.39 (Bolger et al. 2014), with settings including LEADING:10, TRAILING:10, SLIDINGWINDOWS:4:20, and MINLEN:50. Post-trimming, the dataset containing 44,877,348 reads was deposited in the SRR database under the accession number SRR27354904. We then assembled the complete chloroplast genome utilizing GetOrganelle v1.7.1 (Jin et al. 2020). The assembly was annotated through CPGAVAS2 (Shi et al. 2019) and underwent further manual refinement using Apollo (Pontius 2018). The finalized, annotated chloroplast genome was diligently submitted to GenBank, receiving the accession number OQ411035. Additionally, a comprehensive circular genome map was produced based on the outputs from CPGview (Liu et al. 2023).

To establish the phylogenetic relationship of *R. palustris*, the plastome sequences of 22 other species of *Rorippa* were downloaded from GenBank, which exhibited the highest degree of similarity to *R. palustris*. In addition, the outgroups were *Coalisina paradoxa* and *Tarenaya hassleriana*. The entire plastome sequences were aligned using MAFFT software (https://mafft.cbrc.jp/alignment/software/) with default parameters (Katoh and Standley 2016). Following this, a maximum-likelihood (ML) phylogenetic tree was constructed using IQ-TREE (v2.0) (Nguyen et al. 2015) with the Best-fit model of TVM + F + I + G4 along with 1000 bootstrap replicates. To check the reliability of the tree, the researchers used the Shimodaira-Hasegawa (SH) (Shimodaira and Hasegawa 1999) and proximately unbiased (AU) (Shimodaira 2002) methods embedded in IQ-TREE. The primary test script used was iqtree −s input_mafft.phy −m TVM + F + I + G4 −z genome.unconstrain.constrain.treels −zb 10000 −zw −au. To evaluate the divergence of Rorippa plastomes, we calculated the nucleotide diversity using sliding windows with a length of 600 bp and a step size of 200 bp (Cui et al. 2020). Additionally, we compared the cp genomes of R. palustris with three other plastomes using mVISTA in Shuffle-LAGAN mode.

## Results

**Figure 2.**
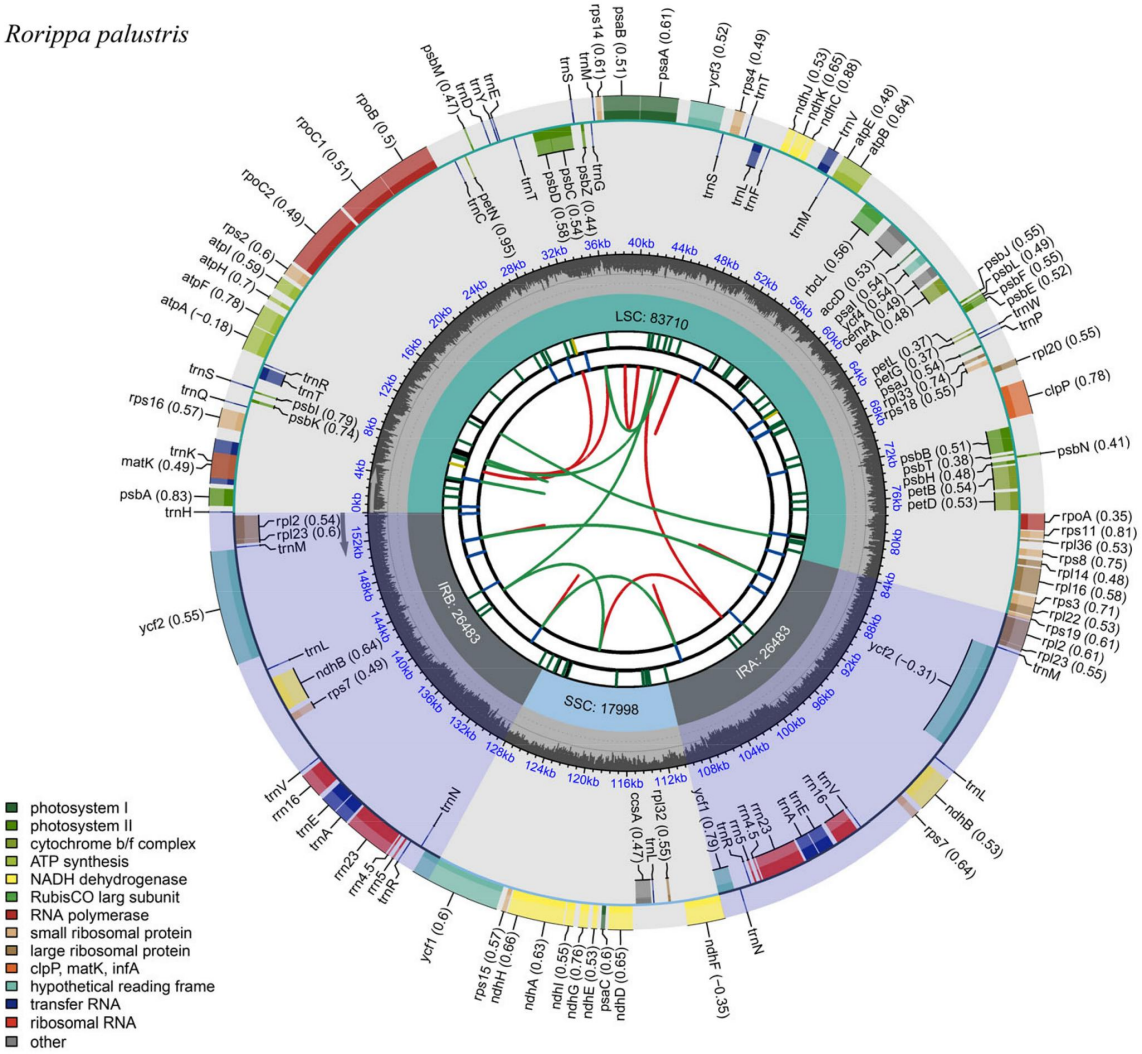
Gene map of the complete plastome genome of *Rorippa palustris*. The species name is shown in the top left corner. The map contains six tracks by default. From the Center outward, the first track shows dispersed repeats, including direct and palindromic repeats connected by red and green arcs. The second track displays long tandem repeats as blue bars, while the third display short tandem repeats or microsatellite sequences as differently colored short bars. These colors correspond to the type and description of each repeat, with black representing complex repeats, green for repeat unit size 1, yellow for size 2, purple for size 3, blue for size 4, orange for size 5, and red for size 6. The fourth track displays the SSC, IRa, IRb, and LSC regions. The fifth track shows the GC content along the genome, while the sixth track sounds the genes. The gene names are followed by optional information about codon usage bias and color-coded based on their functional classification. The inner genes are transcribed clockwise, and the outer genes are transcribed anticlockwise. The available type of the genes is shown in the bottom left corner.

The plastome sequence of *R. palustris* measures 154,674 bp in length and displays a classic quadripartite structure. It consists of two IR regions, each 26,483 bp in length, separated by a large LSC region of 83,710 bp and a small SSC region of 17,998 bp (Figure 2). Mapping experiments demonstrate the high reliability of the genome assembly with the average and minimum depth of 4078.13× and 1185× (Figure S1). The plastome exhibits a varying GC content distribution, with an overall GC content of 36.4%. The highest GC content is found in the IR regions (42.3%), whereas the corresponding values are 34.1% and 29.2% for the LSC and SSC regions, respectively. The *R. palustris* plastome in this study also exhibits an overall GC content of 36.4%. The highest GC content is found in the IR regions (42.4%), whereas the corresponding values are 34.1% and 29.1% for the LSC and SSC regions.

The *R. palustris* plastome was predicted to contain 128 genes, including 83 protein-coding genes (PCGs), eight rRNA genes, and 37 tRNA genes. Six unique PCGs (*rps*12, *rps*7, *rpl*2, *rpl*23, *ndh*B and *ycf*2), seven unique tRNA genes (*trn*A, *trn*E, *trn*L, *trn*M, *trn*N, *trn*R and *trn*V) and four unique rRNA genes (*rrn*16S, *rrn*23S, *rrn*4.5S, *rrn*5S) were located in the IR regions. The presence of the reverse complementary region is conducive to enhancing the stability and conservation of the plastome *of Rorippa*. In the entire plastome, nine PCGs (*rps*16, *atp*F, *rpo*C1, *pet*B, *pet*D, *rpl*16, *rpl*2, *ndh*B, and *ndh*A) and six tRNA genes (*trn*K-UUU, *trn*T-CGU, *trn*L-UAA, *trn*V-UAC, *trn*E-UUC, and *trn*A-UGC) each contain one intron. The structures of the cis-splicing genes are shown in Figure S2. The complete plastome of *R. palustris* did not have a trans-splicing gene. The protein-coding genes, tRNA genes, and rRNA genes in the plastome are 78,702bp, 2,792bp, and 9,050bp in length, accounting for 50.9%, 1.8%, and 5.9% of the total genome length.

**Figure 3.**
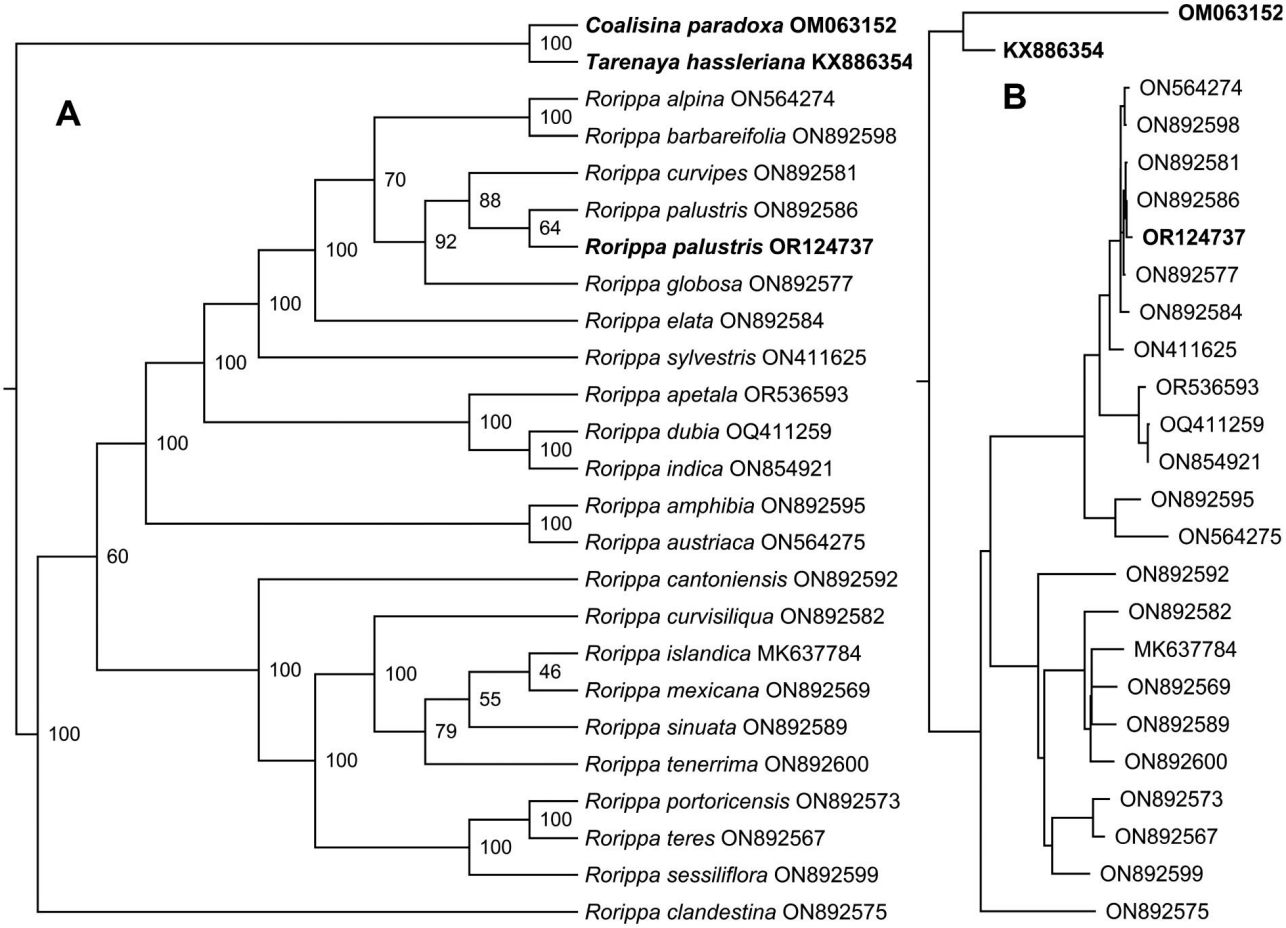
The maximum likelihood phylogeny of *Rorippa palustris* and its close relatives using whole genome sequences. The bootstrap values based on 1000 replicates were shown on each node in the cladogram tree (a). The corresponding phylogram tree is shown in panel B. We downloaded 22 other *Rorippa* species plastome from GenBank, *R. alpina* (ON564274), *R. barbareifolia* (ON892598), *R. curvipes* (ON892581), *R. palustris* (ON892586), *R. globosa* (ON892577), *R. elata* (ON892584), *R. sylvestris* (ON411625) (shao 2022), *R. apetala* (OR536593), *R. dubia* (OQ411259) (Hu and kang 2023), *R. indica* (ON854921) (Wang et al. 2022), *R. amphibia* (ON892595), *R. austriaca* (ON564275), *R. cantoniensis* (ON892592), *R. curvisiliqua* (ON892582), *R. islandica* (MK637784) (rigault et al. 2020), *R. mexicana* (ON892569), *R. sinuata* (ON892589), *R. tenerrima* (ON892600), *R. portoricensis* (ON892573), *R. teres* (ON892567), *R. sessiliflora* (ON892599), *R. clandestina* (ON892575). *Coalisina paradoxa* (OM063152, outgroup) (aljuhani 2022) and *Tarenaya hassleriana* (KX886354, outgroup) (Guo et al. 2017) are served as outgroups labeling in bold font. The new *R. palustris*(OR124737) plastome in this study was labeled in bold font.

The ML phylogenetic tree shows that *R. palustris* is closely related to *R. curvipes*, and a monophyletic clade is formed by two *R. palustris* plants and an *R. curvipes* plant with 88% bootstrap values (Figure 3). Highly variable regions within plastomes offer a rich source of markers for distinguishing closely related species and contribute significantly to phylogenetic studies. In the study, the nucleotide diversity, represented by the pi value, varied between 0 and 0.00111 (Figure S3). Our findings identified four regions with notable variability, which have the potential to serve as molecular markers for species identification within the genus *Rorippa* (Table S1). Although some nodes have relatively low bootstrap values, the topological structure of the ML tree is significantly supported, with a *p*-SH value of 1.00 and a *p*-AU value of 0.995, respectively. Therefore, the phylogenetic analysis provides a reliable account of *R. palustris’* evolution.

## Discussion and conclusion

This study presents the primary characterization of the plastome of *R. palustris* for the first time, which exhibits a typical annular tetrad structure with a size of 154,674 bp and 128 predicted genes. The plastome exhibits a varying GC content distribution in LSC, SSC and IR regions, with an overall GC content of 36.4%. Based on the phylogenetic analysis, *R. palustris* and *R. curvipes* cluster within the Brassicaceae.

The genome structure and base composition of the *R. palustris* plastome analyzed in this study (labeled OR124737) are similar to those of another *R. palustris* plastome (labeled ON892586) (Table S2). Although the plastome in this study (OR124737) contains three fewer predicted genes compared to the other *R. palustris* plastome (ON892586), its total length is only two base pairs longer. When compared with plastomes of other species within the same genus, the structure of the *R. palustris* plastome aligns with the general characteristics typical of the Brassicaceae family (Guo et al. 2017), with only minor differences reported among species (Duan et al. 2015; Guo et al. 2017). Similarly, the average GC content of the *R. palustris* plastome shows minimal variation across different species (Guo et al. 2017).

The plastome, which is maternally inherited, exhibits considerable variability in gene content and genome structure compared to the nuclear genome, with limited recombination events (Du et al. 2020). In this study, we identified a loss of the trans-splicing gene *rps*12 in the *R. palustris* plastome (OR124737), which may be attributed to gene pseudogenization. In contrast, another *R. palustris* plastome (ON892586) retains an intact *rps*12 gene. A similar pattern is observed in other species, such as *R. sessiliflora* and *R. austriaca*. The chloroplast genomes of *R. sessiliflora* (ON892599, ON892588, ON892578) contain an intact *rps*12 gene, whereas the genome labeled ON892587 lacks this gene. Similarly, in *R. austriaca*, the chloroplast genome (ON564277) possesses an intact *rps*12 gene, while the genome labeled ON564276 does not.

These findings suggest that the loss of *rps*12 may be a recurring event in the evolution of *Rorippa* plastomes. Given the role of *rps*12 in ribosomal protein synthesis, its loss could have important implications for plastome function and stability, potentially affecting both transcriptional and translational processes. Future research should aim to investigate the phenotypic effects of these gene deletions and explore potential compensatory mechanisms that may mitigate their impact.

## Data Availability

In this study, the complete plastome genome sequence of *R. palustris* has been submitted to the NCBI database under the accession number OR124737. https://www.ncbi.nlm.nih.gov. The associated BioProject, Bio-Sample, and SRA numbers are PRJNA928567, SAMN38140084, and SRR26700169.
